# Reduced Membrane Insertion of CLC-K by V33L Barttin Results in Loss of Hearing, but Leaves Kidney Function Intact

**DOI:** 10.3389/fphys.2017.00269

**Published:** 2017-05-15

**Authors:** Hua Tan, Stefanie Bungert-Plümke, Christoph Fahlke, Gabriel Stölting

**Affiliations:** Institute of Complex Systems - Zelluläre Biophysik (ICS-4), Forschungszentrum JülichJülich, Germany

**Keywords:** CLC channel, barttin, Bartter syndrome, hearing loss, patch clamp

## Abstract

In the mammalian ear, transduction of sound stimuli is initiated by K^+^ entry through mechano-sensitive channels into inner hair cells. K^+^ entry is driven by a positive endocochlear potential that is maintained by the marginal cell layer of the stria vascularis. This process requires basolateral K^+^ import by NKCC1 Na^+^−2Cl^−^−K^+^ co-transporters as well as Cl^−^ efflux through ClC-Ka/barttin or ClC-Kb/barttin channels. Multiple mutations in the gene encoding the obligatory CLC-K subunit barttin, *BSND*, have been identified in patients with Bartter syndrome type IV. These mutations reduce the endocochlear potential and cause deafness. As CLC-K/barttin channels are also expressed in the kidney, patients with Bartter syndrome IV typically also suffer from salt-wasting hyperuria and electrolyte imbalances. However, there was a single report on a *BSND* mutation that resulted only in deafness, but not kidney disease. We herein studied the functional consequences of another recently discovered *BSND* mutation that predicts exchange of valine at position 33 by leucine. We combined whole-cell patch clamp, confocal microscopy and protein biochemistry to analyze how V33L affects distinct functions of barttin. We found that V33L reduced membrane insertion of CLC-K/barttin complexes without altering unitary CLC-K channel function. Our findings support the hypothesis of a common pathophysiology for the selective loss of hearing due to an attenuation of the total chloride conductance in the stria vascularis while providing enough residual function to maintain normal kidney function.

## Introduction

Barttin constitutes the obligatory β-subunit of two epithelial CLC-type chloride channels, ClC-Ka (also known as ClC-K1 in rodents) and ClC-Kb (ClC-K2 in rodents). It is required for proper membrane targeting, stabilization and activation of these channels (Fahlke and Fischer, 2010; Stölting et al., 2014a). In the kidney, ClC-Ka is the main chloride conduction pathway in the thin ascending limb of Henle's loop (Uchida et al., 1995; Vandewalle et al., 1997) whereas ClC-Kb is expressed in the thick ascending limb of Henle's loop, the distal connecting tubules and in a- and b-intercalated cells of the collecting duct (Vandewalle et al., 1997). Loss-of-function of either channel leads to disturbed kidney function as described in knock-out mouse models for the homologous channels ClC-K1 and ClC-K2 (Matsumura et al., 1999; Grill et al., 2016; Hennings et al., 2016) but also in disease-causing mutations for the human ClC-Kb channel (Simon et al., 1997; Konrad et al., 2000). Both channels are thought to be co-expressed in the stria vascularis of the inner ear so that only the combined loss-of-function results in sensorineural deafness in addition to the renal symptoms (Schlingmann et al., 2004).

Naturally occurring mutations in the gene encoding barttin, *BSND*, cause Bartter syndrome IV that is characterized by impaired urinary concentration and deafness (Bartter et al., 1962; Birkenhäger et al., 2001). Barttin and CLC-K co-localize in the kidney and in the inner ear (Estévez et al., 2001), and most disease-causing mutations of barttin result in a loss of channel function leading to both, renal disease and deafness (Janssen et al., 2009). However, one mutant, I12T barttin, was found to cause deafness, but leave renal function unaffected (Riazuddin et al., 2009). This mutation reduces surface membrane insertion of CLC-K channels so that the total chloride transport capacity of affected epithelia is attenuated, but not abolished. Based on these findings, it was hypothesized that the inner ear is more sensitive to a reduced chloride conductance than the loop of Henle (Riazuddin et al., 2009; Fahlke and Fischer, 2010). Recently, another mutation in barttin, V33L, was associated with deafness without impaired kidney function in a Pakistani family (Shafique et al., 2014) but not studied on a molecular level so far.

We applied a combination of whole-cell patch clamp, surface biotinylation and confocal microscopy to study the effect of the V33L barttin mutation on the function and trafficking of ClC-Ka and ClC-Kb in order to further elucidate the mechanism behind barttin mutations selectively impairing hearing but not kidney function.

## Methods

### Construction of expression plasmids, mutagenesis, and heterologous expression

Coding regions of barttin, ClC-Ka or ClC-Kb were cloned into pcDNA3.1, pcDNA5/FRT/TO or pRc/CMV vectors (Life Technologies), with eGFP or mVenus linked to the amino-terminus of the channels and mCherry to the carboxy-terminus of barttin. Previous publications showed no effects of this procedure on expression or function of the channel or its accessory subunit (Scholl et al., 2006; Janssen et al., 2009; Fischer et al., 2010). The V33L mutation was introduced into barttin by overlapping extension PCR.

We heterologously expressed WT and mutant CLC-K/barttin channels in two different cell lines, MDCK II and HEK293T cells. MDCK II cells are known to show epithelial properties, such as cell polarization and proper sorting and trafficking (Cereijido et al., 1978), and we therefore employed confluent MDCK II cells for studies of channel localization and trafficking. However, MDCK II cells exhibit significant background currents in addition to loss of polarization upon cell dispersion which is required for whole-cell patch clamping. We performed the electrophysiological characterization of CLC-K/barttin in HEK293T cells. These cells do not resemble polarized epithelia as well as MDCK II cells, but are well suited for electrophysiological experiments because of negligible background currents and robust expression of heterologous proteins.

HEK293T cells were co-transfected with 1 μg of pcDNA3.1 eGFP- or mVenus-ClC-Ka/-Kb and 3 μg of pcDNA3.1 barttin-mCherry (or barttin-V33L-mCherry) using the calcium phosphate technique (Fahlke et al., 2001) in a 5-cm petri dish with 3 mL growth medium. The cells were split 20 h after transfection and used for patch clamp recording the next day after splitting. For co-immunoprecipitation experiments, cells were washed with PBS supplemented with 0.05% (w/v) EDTA 20 h after transfection and harvested immediately afterwards.

MDCK II cells were transfected using Lipofectamine 2000 (Life Technologies) according to the supplied protocol, with 1.5 μg pcDNA3.1 eGFP- or mVenus-ClC-Ka/-Kb and/or 1.5 μg pcDNA3.1 barttin-mCherry (or barttin-V33L-mCherry) in a 3.5-cm ibiTreat μ-dish for the confocal imaging, or with 4 μg pcDNA3.1 eGFP- or mVenus-ClC-Ka/-Kb and/or 4 μg pcDNA3.1 barttin-mCherry (or barttin-V33L-mCherry) in a 10-cm petri dish for biotinylation. After transfection cells were grown for 1 to 2 days to reach a polarized, confluent state for confocal scanning.

### Whole-cell patch clamp and fluorescence-current correlation

Whole-cell patch clamp recordings were performed using an AxoPatch 200B amplifier controlled by pClamp software (Molecular Devices) (Hebeisen and Fahlke, 2005) or a HEKA EPC-10 (HEKA Elektronik) as previously described (Stölting et al., 2014b) Recordings were filtered using a 10 kHz lowpass Bessel filter. The external solution contained (in mM): 140 NaCl, 4 KCl, 2 CaCl_2_, 1 MgCl_2_, and 10 HEPES while the internal solution contained 115 NaCl, 2 MgCl_2_, 5 EGTA, and 5 HEPES. Both solutions were adjusted to pH 7.4. In experiments combining fluorescence measurements and whole-cell recordings the patch clamp mode was established as described above, and eGFP was excited using a Polychrom V monochromator (TILL Photonics) set to 488 nm and recorded using a Neo camera (Andor) (Schänzler and Fahlke, 2012). Total fluorescence intensities for manually selected cells of interest were analyzed using Fiji (http://www.fiji.sc).

### Noise analysis

CLC channels are double-barreled, with two identical “protopores” that are opened and closed by individual as well as by common gating processes (Miller and White, 1984; Ludewig et al., 1996; Middleton et al., 1996; Stölting et al., 2014b). For such a channel, the whole cell current amplitude (*I*) depends on the number of channels in the surface membrane (*N*), the single channel pore amplitude (*i*) and the open probabilities of the two protopore gates (*P*_*p*_) and the common gate (*P*_*c*_) (Accardi and Pusch, 2000; Fischer et al., 2010):

(1) $$\begin{array}{l} I=2N\cdot i\cdot P_{p}\cdot P_{c} \end{array}$$

Random opening and closing of ion channels produce a Lorentzian type of noise (σ^2^) that depends on the number of channels as well as its unitary current amplitude and its open probability. For a double-barreled channel σ^2^ can be calculated as Fischer et al. (2010), Weinberger et al. (2012) and Stölting et al. (2014a):

(2) $$\begin{array}{l} \sigma^{2}=i\cdot I-\frac{I^{2}}{N}\left(1-\frac{1}{2P_{c}}\right) \end{array}$$

In the case of ClC-Ka/barttin channels the common gate was found permanently open using single channel recordings and noise analysis (Fischer et al., 2010), thus simplifying (2) to

(3) $$\begin{array}{l} \sigma^{2}\;=\;i\cdot I\;-\;\frac{I^{2}}{2N} \end{array}$$

which is equivalent to:

(4) $$\begin{array}{l} \frac{\sigma^{2}}{I}\;=\;i\;-\;\frac{I}{2N} \end{array}$$

Based on an ohmic current-voltage relationship, this relationship can be further simplified to:

(5) $$\begin{array}{l} \frac{\sigma^{2}}{I\cdot \left(V\;-\;V_{rev}\right)}\;=\;\gamma \;-\;\frac{I}{2N\cdot \left(V\;-\;V_{rev}\right)} \end{array}$$

here with *V* the applied voltage, *V*_*rev*_ the reversal potential, *N* the number of functional channels in the surface membrane and γ the single channel pore conductance (Sesti and Goldstein, 1998). The voltage-independent background noise was recorded at the reversal potential, where ClC-Ka/barttin currents do not contribute, averaged and subtracted from the otherwise measured variance.

Unitary channel parameters of ClC-Kb/barttin have so far not been described on a detailed single channel level. The homologous ClC-K2 channel appears to exhibit properties that are incompatible with whole-cell recordings of ClC-Kb (Pinelli et al., 2016) and may not be used for comparison. We therefore estimated unitary properties using a modified noise analysis according to a method described in Stölting et al. (2015). For a regular CLC-type channel with two conduction pathways exhibiting protopore as well as common gating mechanisms (Fischer et al., 2010; Stölting et al., 2014b), combining Equations (1) and (2) results in

(6) $$\begin{array}{l} \frac{\sigma^{2}}{I}=i\cdot \left(1\;-\;P_{p}\left(2P_{c}\;-\;1\right)\right) \end{array}$$

The ratio of variance to the mean macroscopic current depends on the product of single channel amplitudes and open probabilities of protopore and common gates, but is indifferent to variations in the number of channels.

### Confocal imaging

Confocal imaging was performed on living MDCK II cells bathed in standard external solution on an inverted confocal laser scanning microscope (Leica TCS SP5, Leica) using a 63 × /1.4 oil immersion objective. Imaging channels were scanned sequentially to avoid cross-contamination with the co-expressed fluorescent protein. eGFP was excited using a 488 nm laser and emission was collected between 490 and 580 nm, mVenus was excited at 514 nm and detected between 490 and 560 nm, and mCherry was excited using a 543 nm Laser and detected between 600 and 713 nm.

### Biochemical analysis

Co-immunoprecipitation of barttin and human CLC-K channels was performed as described previously (Stölting et al., 2015). Transfected HEK293T cells were collected from petri dishes and lysed using ComplexioLyte-47a (Logopharm). Cleared lysates were incubated with 1 μg of monoclonal anti-GFP antibody (Life Technologies) or left as a control without antibody. Antibody-bound protein complexes were purified using protein G-sepharose beads (Thermo Scientific) and eluted using SDS loading buffer. Samples were run on a 10% SDS gel before scanning.

Cell surface expression of CLC-K channels was investigated using a modification of cell surface biotinylation methods described previously (Nothmann et al., 2011; Stölting et al., 2015; Wojciechowski et al., 2015). MDCK II cells were incubated with 1 mg of EZ linked NHS-Sulfo-SS-biotin (Thermo Scientific) in PBS for 30 min. After quenching of free biotin with 50 mM glycin in PBS for 30 min the cells were lysed using RIPA buffer and incubated with NeutrAvidin beads (Thermo Scientific) for 1 h. Biotinylated proteins were eluted off the column with 2 × SDS sample buffer and run on a 10% SDS gel. Cells transfected with cytosolic mVenus were used as control to test the specificity of this biotinylation protocol to label surface membrane proteins.

Gels were scanned on a fluorescence gel scanner (Typhoon FLA 9500, GE Healthcare) at 100 μm resolution. eGFP and mVenus were excited at 473 nm, and the emission recorded using a 530/20 bandpass filter. The signal of mCherry was recorded using a 532 nm laser and a longpass 575 nm filter. Gel images were quantified using the Fiji software. Gels were rotated up to 3° using bilinear interpolation and subsequently quantified using the built-in tools for gel analysis.

### Statistical analysis

Unless noted otherwise, Student's *t*-test was used for statistical comparison with ^*^ denoting *p* < 0.05, ^**^p < 0.01 and ^***^p < 0.001. We chose a significance level of 5% prior to analysis and therefore did not reject the null hypothesis for comparisons yielding a *p*-value larger than 0.05 (labeled: “n.s.”). All errors are given as s.e.m.

## Results

### V33L barttin reduces whole-cell current amplitudes of CLC-K channels

**Figure 1:**
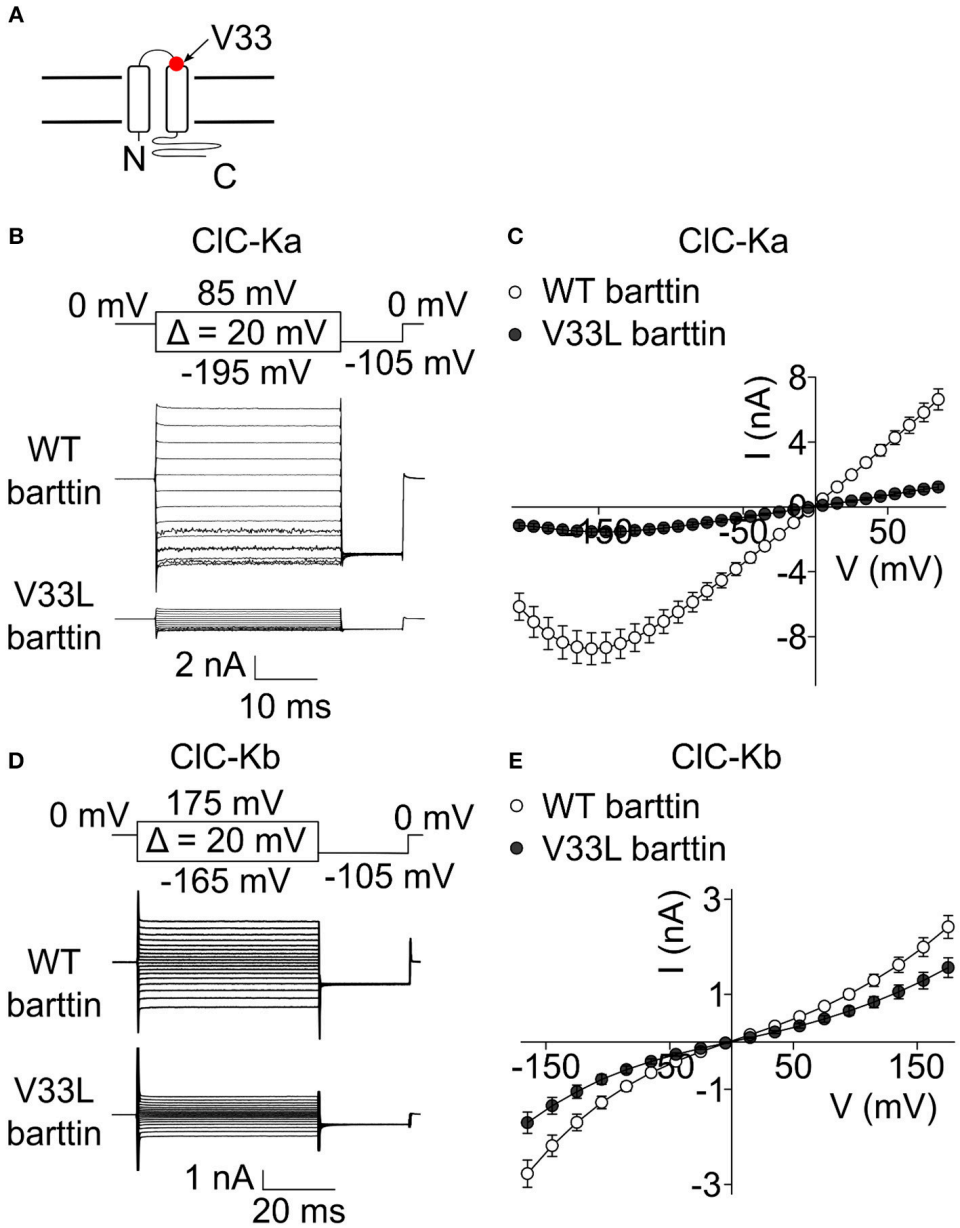
**V33L barttin reduces macroscopic currents of CLC-K channels. (A)** Transmembrane topology of barttin with the position of V33L indicated by an arrow. **(B)** Representative current recordings from HEK293T cells co-expressing ClC-Ka with WT or V33L barttin. **(C)** Voltage-dependence of steady-state currents in cells co-expressing ClC-Ka with WT (*n* = 16) or V33L barttin (*n* = 10). **(D)** Representative current recordings from HEK293T cells co-expressing ClC-Kb with WT or V33L barttin. **(E)** Voltage-dependence of steady-state currents in cells co-expressing ClC-Kb with WT (*n* = 50) or V33L barttin (*n* = 48). All error bars indicate s.e.m.

A recent publication (Shafique et al., 2014) reported a novel *BSND* mutation in deaf patients that predicts the exchange of valine to leucine at position 33 of barttin. V33 is located in the short extracellular loop close to the beginning of the second transmembrane domain of barttin (Figure 1A). To study the consequences of V33L barttin on chloride channel function we co-expressed WT and mutant barttin with ClC-Ka or ClC-Kb in HEK293T cells and measured whole-cell currents with the patch clamp technique. ClC-Ka/barttin displays a linear current-voltage relationship at voltages between −100 mV and +100 mV. At more negative potentials, the whole-cell conductance is gradually decreasing, resulting in a “hook” at approximately −150 mV (Figures 1B,C). ClC-Kb/barttin currents exhibit a characteristic bi-directional rectification (Figures 1D,E). ClC-Kb/barttin whole-cell current amplitudes are smaller than for ClC-Ka/barttin (Figures 1C,E), presumably due to smaller unitary amplitudes as reported for their rodent homologs (Fischer et al., 2010; Pinelli et al., 2016). V33L barttin left the time and voltage dependence of ClC-Ka/barttin and ClC-Kb/barttin currents unaltered (Figures 1B–E). ClC-Ka/V33L barttin currents, however were reduced to <20% (*p* < 0.001 at −155 mV; Figures 1B,C). The differences between ClC-Kb/WT barttin and ClC-Kb/V33L barttin currents were only slightly decreased (*p* = 0.004 at −165 mV; Figures 1D,E).

### Unitary properties of ClC-Ka/barttin and ClC-Kb/barttin are unaffected by the V33L mutation

The reduction in whole-cell CLC-K currents in the presence of V33L barttin might either be caused by a reduction of the open probability, diminished single channel amplitude or by a reduced number of channels in the surface membrane. Since ClC-Ka/barttin channels exhibit a run-down in excised patches, and the gating properties of these channels make estimation of unitary channel properties difficult in the cell-attached mode (Fischer et al., 2010), we determined ClC-Ka/barttin single channel properties using a modified stationary noise analysis that has previously shown similar results to single channel recordings also for other ion channels (Sesti and Goldstein, 1998; Fischer et al., 2010; Stölting et al., 2013).

**Figure 2:**
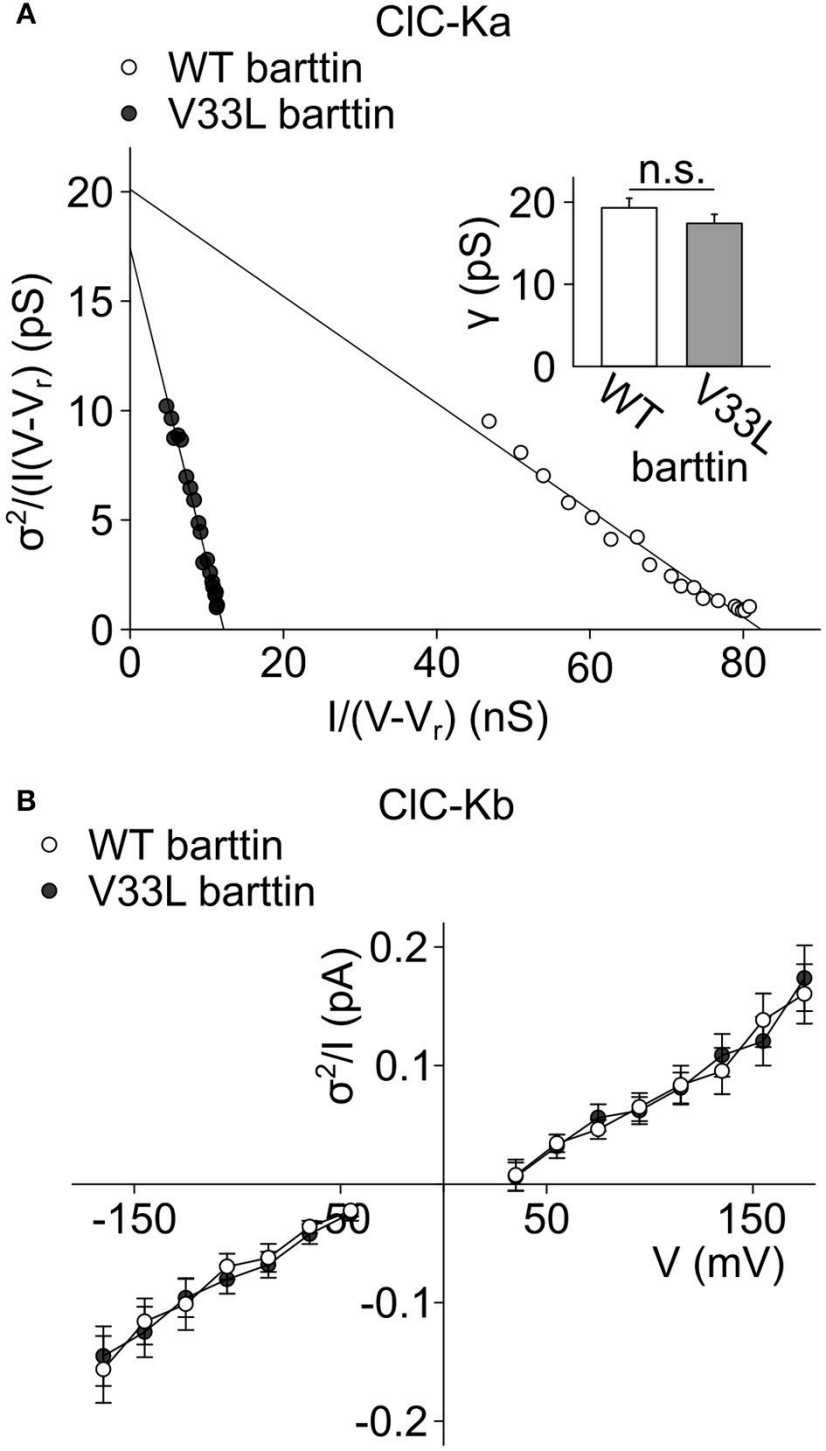
**V33L barttin leaves single channel properties of ClC-Ka/barttin and ClC-Kb/barttin unaffected. (A)** Representative noise analyses from cells co-expressing ClC-Ka with either WT (open circles) or V33L barttin (filled circles). The y-axis intercept provides the unitary conductance and the slope of the fitted line the number of protopores according to Equation 5. Inset, Mean values indicate similar unitary protopore conductances for ClC-Ka/barttin with WT or V33L barttin (V33L: *n* = 8, WT: *n* = 13, *p* = 0.3). **(B)** Voltage dependence of current variance by amplitude ratios for ClC-Kb/barttin with WT or V33L barttin according to Equation 6 (V33L: *n* = 34, WT: *n* = 41, *p* = 0.7 at −165 mV).

We determined steady-state current amplitudes and variances for protocols similar to the ones shown in Figure 1 and plotted variances divided by the product of the mean current and the driving force (*V* − *V*_*rev*_) vs. the mean current divided by the driving force (Equation 5) (Figure 2A). A linear fit to these values yields the unitary protopore conductance as y-axis intercept, and the slope of the linear regression corresponds to the number of active protopores in the plasma membrane or the twice the number of channels (*N*) according to:

(7) $$\begin{array}{l} N\;=\;\frac{-1}{2\cdot slope} \end{array}$$

Absolute open probabilities were calculated according to Equation 1 after obtaining protopore open probabilities by dividing current amplitudes by the protopore current amplitude i and N. We obtained unaltered unitary conductances (Figure 2A, inset) as well as absolute open probabilities: For both WT (*n* = 13) and V33L (*n* = 8), the single ClC-Ka/barttin channel pore opens in a probability range from 0.5 at −195 mV to almost 1 at voltages positive to −105 mV under the recording conditions. The difference in the slopes of the distributions demonstrates that V33L reduces the number of ClC-Ka channels (for WT 1944 ± 307, *n* = 13; for V33L 444 ± 51, *n* = 8).

This noise analysis is based on two assumptions, i.e., that the common gate is permanently open and that the current-voltage relationship for unitary channels is linear. These assumptions have been experimentally tested for WT ClC-Ka/barttin by single channel recordings (Fischer et al., 2010). For the homologous mouse ClC-K1/barttin channel, however, there has been an alternative interpretation in that the protopore gates are constitutively open and only common gating occurs (L'Hoste et al., 2013). Even in that case, the noise analysis applied to our data is still valid, although unitary current amplitudes would then correspond to openings and closures of both protopores simultaneously. Furthermore, the time and voltage dependence of macroscopic ClC-Ka/barttin currents is not affected by the V33L mutation, indicating that the mutation does not affect the current-voltage relationship. A change in slow gating would result in the occurrence of channels with two subconductance states (Miller and White, 1984). Noise analysis on currents generated by such channels produce apparent unitary current amplitudes intermediate to the single protopore and the full dimeric double protopore conductance levels (Weinberger et al., 2012; Stölting et al., 2014a). Such a change in apparent unitary current amplitude was not observed in our experiments.

Whereas the effect of V33L on unitary channel properties of ClC-Ka/barttin could be determined with noise analysis, we could not obtain unitary current amplitudes and absolute open probabilities of ClC-Kb/barttin channels. These channels display only small voltage-dependent changes of the open probability within the tested range. Moreover, absolute values for protopore or common gate open probability are not known, precluding the use of noise analysis for determining unitary current properties of ClC-Kb/barttin. We compared ratios of ClC-Kb/barttin current variances by current amplitudes as described previously (Stölting et al., 2015). This ratio (Equation 6) depends on the open probabilities of the protopore as well as the common gate and the single channel amplitude. This value was found to be unchanged in recordings with WT and V33L barttin, strongly supporting the notion that single channel properties are unchanged by V33L barttin also for ClC-Kb/barttin (Figure 2B). We conclude that V33L reduces the number of ClC-Ka/barttin and ClC-Kb/barttin channels in the surface membrane of HEK293T cells.

### V33L does not impair association of barttin with CLC-K

**Figure 3:**
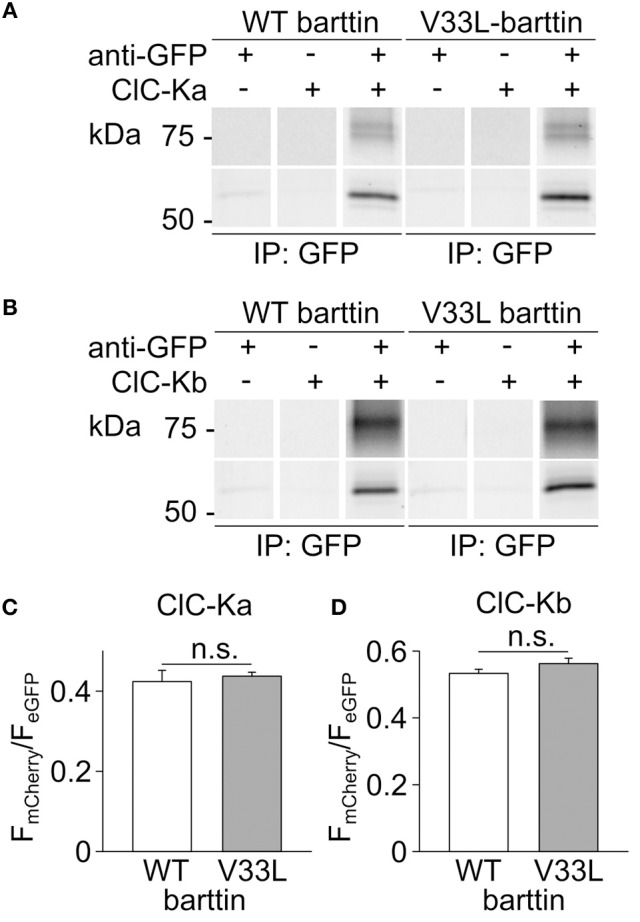
**V33L does not affect association of barttin to ClC-Ka or ClC-Kb. (A,B)** Representative fluorescence scans of SDS-PAGE gels demonstrate the co-purification of WT or V33L barttin-mCherry with an anti-GFP antibody targeting either eGFP-ClC-Ka **(A)** or eGFP-ClC-Kb **(B)**. **(C,D)** Ratios of band intensities for co-purified barttin-mCherry to the intensity of the eGFP-ClC-Ka **(C)** or ClC-Kb **(D)** are similar for WT and V33L barttin (ClC-Ka: *n* = 4 each, *p* = 0.7; ClC-Kb: *n* = 4 each, *p* = 0.2).

The observed reduction in the number of membrane-inserted CLC-K/barttin channels by V33L barttin may arise from impaired CLC-K/barttin-interaction and reduced stability of the CLC-K/barttin complex. We performed co-immunoprecipitation experiments to test whether V33L decreases the affinity of barttin binding to CLC-K. HEK293T cells were co-transfected with either eGFP tagged ClC-Ka or ClC-Kb and WT or V33L barttin-mCherry. After lysis and solubilization of membrane proteins anti-GFP antibodies were used to link eGFP-tagged CLC-K channels to protein G agarose beads. WT and V33L barttin bound to CLC-K could be co-eluted from the beads and identified using fluorescence scans of SDS-PAGE gels (Figures 3A,B).

As previously described, barttin co-expression leads to significant complex glycosylation of CLC-K channels (Waldegger et al., 2002; Janssen et al., 2009). The intensity ratios of the respective barttin bands over the summed intensities of the channel bands at all glycosylation-states were similar for WT and V33L barttin and both CLC-K channels, respectively (Figures 3C,D). These results indicate that V33L does not reduce the number of CLC-K/barttin channels in the surface membrane by impairing barttin-CLC-K interactions.

### V33L barttin impairs trafficking of ClC-Ka and ClC-Kb

Reduced whole-cell current amplitudes together with unchanged unitary channel parameters and unchanged CLC-K-barttin binding strongly indicate that V33L impairs surface membrane insertion of CLC-K/barttin channels. To quantify these changes mediated by V33L barttin in HEK293T cells, we combined whole-cell patch clamp with fluorometry (Schänzler and Fahlke, 2012; Ronstedt et al., 2015).

The total number of CLC-K subunits—in intracellular organelles as well as in the surface membrane—can be determined by measuring whole-cell eGFP fluorescence. The fluorescence intensity for a single cell (*F*) expressing a GFP-tagged channel can be calculated according to:

(8) $$\begin{array}{l} F\;=\;2N\cdot f \end{array}$$

with *N* being the total number of CLC-K channels in the cell and *f* being the mean fluorescence intensity of a single GFP-CLC-K protein. The mean steady-state macroscopic current (*I*) is determined as:

(9) $$\begin{array}{l} I\;=\;2N\cdot R_{m}\cdot R_{bar}\cdot i\cdot P_{o} \end{array}$$

with *R*_*m*_ being the percentage of channels in the surface membrane; *R*_*bar*_ the percentage of channels bound to barttin; *i* the single channel amplitude and *P*_*o*_ the open probability of a single channel.

Dividing mean macroscopic current amplitudes by the fluorescence intensity results in

(10) $$\begin{array}{l} \frac{I}{F}\;=\;\frac{R_{m}\cdot R_{bar}\cdot i\cdot P_{o}}{f} \end{array}$$

Single channel current amplitudes *i* and open probabilities *P*_*o*_ were shown to be identical for WT and mutant barttin, and the fluorescence of a single GFP-CLC-K protein is expected not to be changed by V33L barttin. Co-immunoprecipitation results suggested a similar affinity so that *R*_*bar*_ can be treated as constant. Ratios of whole-cell current amplitudes and fluorescence for CLC-K with either WT or V33L barttin thus provide the relative membrane insertion probabilities.

**Figure 4:**
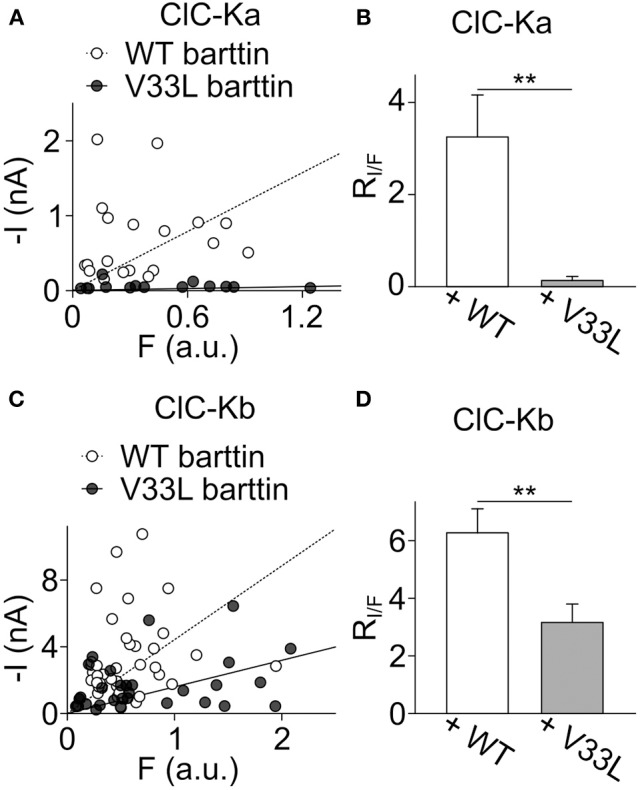
**V33L barttin reduces the ratio of whole-cell currents to eGFP-fluorescence intensities. (A)** Plot of the steady-state current at −75 mV vs. whole-cell fluorescence for cells co-expressing ClC-Ka with WT (open circles) or V33L barttin (filled circles). **(B)** Mean current-fluorescence ratios are significantly lower in cells co-expressing ClC-Ka with V33L barttin (*n* = 14) than with WT barttin (*n* = 19, *p* = 0.003) indicating lower membrane insertion with V33L barttin. **(C)** Correlation between steady-state current at −175 mV and whole-cell fluorescence for cells co-expressing ClC-Kb with WT (open circles) or V33L barttin (filled circles). **(D)** Mean current-fluorescence ratios are significantly lower in cells co-expressing ClC-Kb with V33L barttin (*n* = 31) than with WT barttin (*n* = 40, *p* = 0.004).

Plotting *I* against *F* yielded an overlapping distribution of values (Figures 4A,C), however, fitting linear functions provided mean current/fluorescence ratios that were significantly smaller for ClC-Ka and ClC-Kb in the presence of V33L barttin than with WT barttin (Figures 4B,D). We conclude that V33L reduces surface membrane insertion as the underlying cause of the experimentally observed attenuation of CLC-K/barttin currents.

### V33L barttin impairs plasma membrane insertion of ClC-Ka and ClC-Kb

HEK293T cells differ from epithelial cells in their inability to form polarized epithelia. However, trafficking and sorting of transmembrane proteins into either apical or basolateral plasma membranes is a hallmark of epithelia and crucial for directed transport of solutes across the epithelial layer. Since MDCK II cells represent an established model for studies of epithelial proteins in general and CLC-K/barttin complexes in particular, we determined the localization of CLC-K and barttin fusion proteins in transfected MDCK II cells.

**Figure 5:**
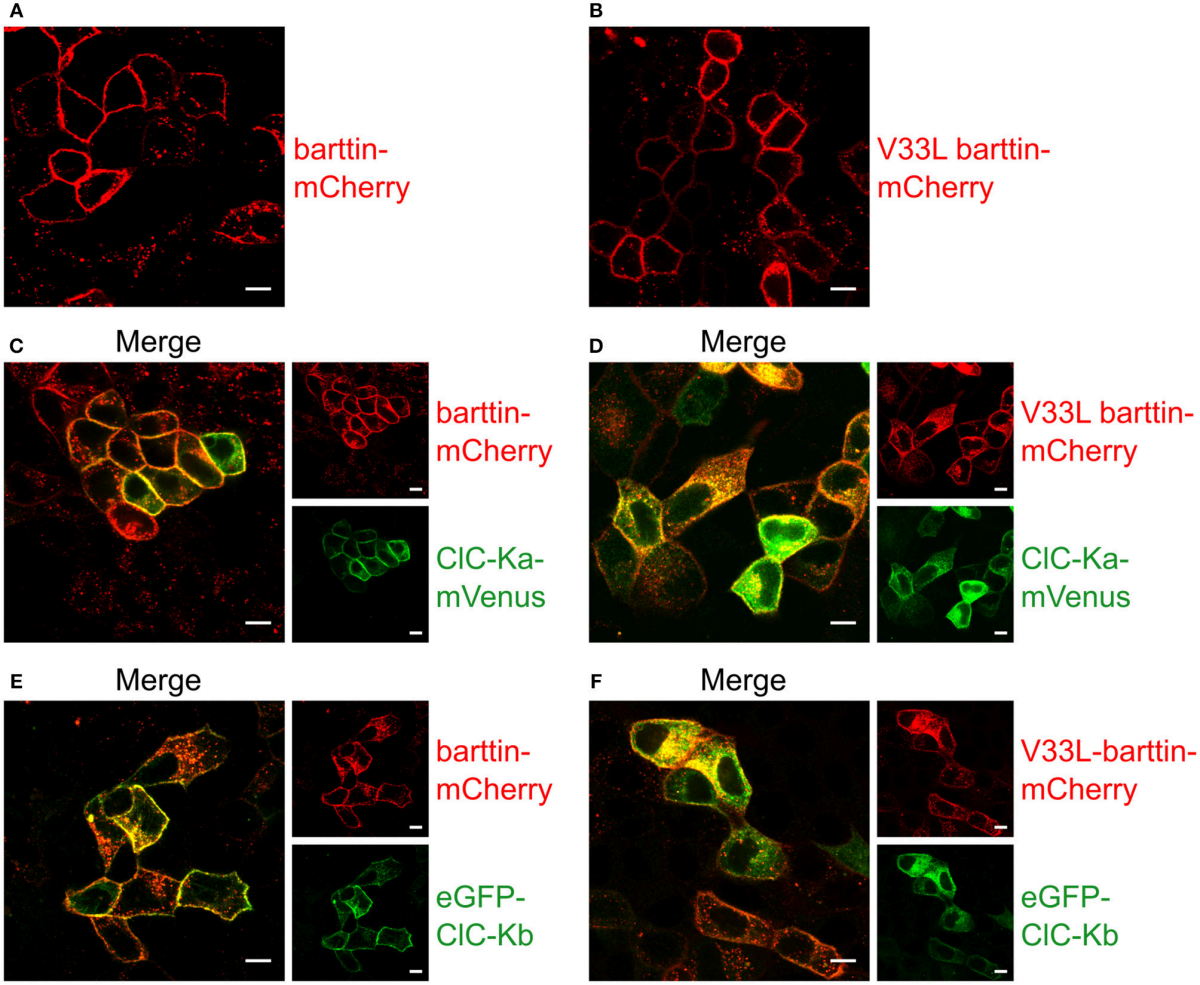
**Representative confocal images of living MDCK II cells confirm a lower membrane insertion of CLC-K channels when co-expressed with V33L barttin. (A,B)** MDCK II cells expressing WT **(A)** or V33L barttin-mCherry **(B)** show a clearly visible membrane staining. **(C,D)** MDCK II cells co-expressing WT or V33L barttin-mCherry with ClC-Ka mVenus show a more clearly delineated membrane staining for ClC-Ka co-expressed with WT barttin **(C)** than with V33L barttin **(D)**. **(E,F)** MDCK II cells co-expressing either barttin with ClC-Kb demonstrate a higher abundance of intracellular ClC-Kb channels when co-expressed with V33L barttin **(F)**.

When expressed alone, WT and V33L barttin, were predominantly inserted into the surface membrane (Figures 5A,B). Without barttin, ClC-Ka and ClC-Kb are retained in the endoplasmic reticulum (Scholl et al., 2006) while co-expression of their accessory subunit significantly increases the number of channels in the plasma membrane. The V33L mutation attenuated surface membrane insertion of ClC-Ka and ClC-Kb. When expressed together with WT barttin, most of ClC-Ka was inserted into the plasma membrane (Figure 5C). Co-expression with V33L barttin, however, resulted in a stronger perinuclear staining of eGFP-ClC-Ka in good agreement with a retention of channels in the endoplasmic reticulum (Figure 5D). Whereas WT barttin effectively brought ClC-Kb into the surface membrane, there was more intracellular staining of cells co-expression ClC-Kb with V33L barttin (Figures 5E,F). The finding that co-expression of V33L barttin with CLC-K channels also results in a more dominant intracellular staining of barttin itself suggests that mutant barttin is retained intracellularly in a complex with CLC-K channels.

**Figure 6:**
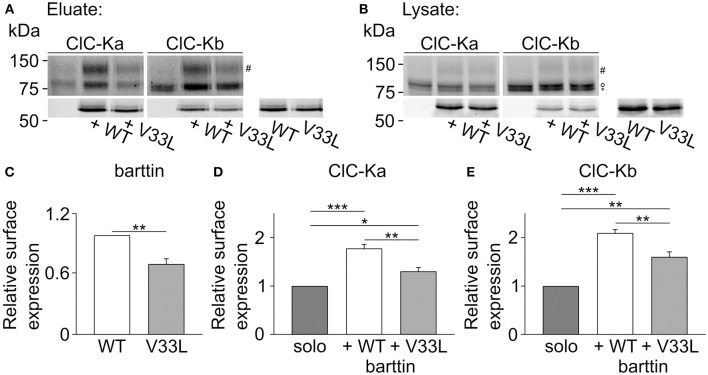
**V33L barttin reduces the membrane insertion of ClC-Ka and ClC-Kb in MDCK II cells. (A,B)** Representative fluorescence scan of a SDS-PAGE gel from the purified fraction after surface biotinylation (**A**, eluate) or the whole cell lysate (**B**, lysate). The upper panels depict eGFP-ClC-Ka or eGFP-ClC-Kb in different glycosylation states (“#” complex-glycosylated, “°” core-glycosylated, and ^“*”^ unglycosylated). The lower panels show WT or V33L barttin-mCherry. **(C–E)** Relative surface expression calculated as ratio of eluate to whole cell lysate intensity for barttin expresed alone (*n* = 6, *p* = 0.004), of ClC-Ka (**D**, *n* = 6) or ClC-Kb (**E**, *n* = 6) co-expressed with WT or V33L barttin.

We next performed surface biotinylation on transiently transfected MDCK II cells to quantify those changes in surface membrane expression. Proteins in the plasma membrane were labeled and purified, and the subsequent quantification of protein in the surface membrane and total protein provides a quantitative measure of surface membrane insertion probability. Figure 6 depicts representative fluorescence scans of SDS-PAGE gels from the biotinylated fraction (Figure 6A) and the whole cell lysate fraction (Figure 6B). Ratios of biotinylated protein by full lysates were smaller for V33L than for WT barttin (Figure 6C), for ClC-Ka as well as for ClC-Kb (Figures 6D,E), further supporting the notion that V33L reduces surface membrane insertion of ClC-Ka and ClC-Kb. Whereas the difference in whole cell current amplitudes was not as pronounced for ClC-Kb as for ClC-Ka, the change in membrane insertion as determined by surface biotinylation was comparable for the two pore-forming subunits (Figures 6D,E). This difference between expression systems suggests that cell-type specific differences in ClC-K/barttin trafficking between HEK293T and MDCK II cells. One might speculate that the two channels utilize different trafficking pathways at least in HEK293T cells and that trafficking of CLC-K channels also depends on factors other than barttin.

## Discussion

Naturally occuring *BSND* mutations can result in sensorineural deafness and salt-losing polyuria (Birkenhäger et al., 2001). Many of these disease-associated mutations are either nonsense or missense mutations which typically abolish channel activation resulting in loss-of-chloride channel function (Janssen et al., 2009). There is, however, an earlier report about the missense mutation I12T barttin that only causes deafness leaving kidney function unaffected (Riazuddin et al., 2009). In heterologous expression systems I12T only mildly affects trafficking of CLC-K/barttin channels, indicative of a decreased, but still persisting chloride conductance in affected epithelia. As the affected mutation carriers only suffered from deafness, but not impaired kidney function it was proposed that either the remaining epithelial CLC-K/barttin conductance is sufficient to prevent salt-losing polyuria or that other mechanisms might compensate for the loss of CLC-K/barttin in renal epithelia. We herein studied the functional consequences of another naturally occurring *BSND* mutation that was reported to selectively cause deafness without renal symptoms, V33L barttin (Shafique et al., 2014). We demonstrate that V33L impairs surface membrane insertion of both ClC-Ka and ClC-Kb, (Figures 4, 6), but leaves unitary currents and absolute open probabilities of ClC-Ka/barttin and ClC-Kb/barttin unaltered (Figure 2). These changes will result in a significant reduction of epithelial chloride conductance, but not in a complete loss-of-function.

A recent computational analysis of inner ear function predicts a significant drop of the endocochlear potential upon reduction in the baso-lateral chloride conductance in marginal cells of the stria vascularis (Nin et al., 2012). The reason for this drop in endocochlear potential is that - in the absence of functional CLC-K channels - chloride accumulates, preventing further uptake of potassium into marginal cells by NKCC1 (Nin et al., 2008; Rickheit et al., 2008). There exists only a small gradient of the chloride concentration across the baso-lateral membrane in marginal cells with slightly higher concentrations inside marginal cells than in the interstriatal space (Ikeda and Morizono, 1989; Nin et al., 2012). Similarily, the membrane potential across the baso-lateral membrane of marginal cells is close to 0 mV (Salt et al., 1987) suggesting only a small constitutive outward transport of chloride ions out of the marginal cells into the intrastriatal space via CLC-K/barttin facilitating the recirculation of chloride across the baso-lateral membrane. Within this assumed physiologically relevant voltage range, our electrophysiological characterization of CLC-K/barttin channels suggests a higher conductance of ClC-Ka over ClC-Kb due to the bidirectional rectification of the latter (Figure 1). ClC-Ka might thus be more significant for the maintenance of the endocochlear potential than ClC-Kb. However, ClC-Kb must be able to compensate for a loss of ClC-Ka as the sole loss of ClC-Ka does not lead to deafness as indicated by a large cohort (Cappola et al., 2011). Additionally, both reported deafness causing mutations in barttin, I12T and V33L show a higher impact on currents through ClC-Ka than ClC-Kb (Figure 1) warranting further investigations into the significance of either isoform in mammalian hearing.

Our results predict a reduction of the total chloride current by 50% up to 80% as based on the reduction in surface expression in MDCK II cells or our current recordings from HEK293T cells. However, in the computational model a 50-fold reduction in conductance is required to cause a significant drop in the endocochlear potential. There might be additional factors that were not incorporated in the computational model (Nin et al., 2012), or V33L might exert more pronounced effects in native cells than in mammalian cells overexpressing CLC-K/barttin.

Even though animal models with a loss of *Clcnk1* (Matsumura et al., 1999) or *Clcnk2* (Grill et al., 2016; Hennings et al., 2016) have been generated, none of these animals was thoroughly tested for hearing impairment. However, one of the *Clcnk2-/-* mice was reported not to display obvious signs of deafness (Grill et al., 2016). It will be an important task for the future to test and to compare the impact of the loss of either ClC-K1 or -K2 on hearing and the generation of the endo-cochlear potential.

Our analysis of functional consequences of the V33L mutation, which selectively affects hearing thresholds, strengthens the idea that inner ear function is more sensitive to the surface expression of CLC-K/barttin than previously believed. Given that the stria vascularis is not protected from systemic circulation by the blood-cochlear-barrier (Jahnke, 1975) this implies that great care has to be taken when considering CLC-K blockers as potential diuretic drugs in the treatment of hypertension (Imbrici et al., 2014; Liantonio et al., 2016). On the other hand, this peculiarity makes the systemic application of activators of CLC-K/barttin (Zifarelli et al., 2010) or drugs enhancing folding and trafficking of barttin (Nomura et al., 2013) for treating sensorineural deafness possible.

Our data together with earlier results support a good correlation between barttin dysfunction and severity of the clinical phenotype in *BSND*-associated diseases (Fahlke and Fischer, 2010). Whereas mutations that abolish barttin expression result in a severe clinical course with early end-stage renal failure, no renal failure occurred in patients carrying barttin mutations that do not completely prevent channel insertion into the surface membrane. Mutations that preserve CLC-K/barttin function cause deafness, but do not affect renal salt extrusion.

## Author contributions

CF and GS conceived and designed the study. All authors contributed to the design of the individual experiments. HT and SB performed experiments. HT, SB, and GS were responsible for data analysis. CF and GS drafted the article, and it was critically revised and finally approved by all authors.

### Conflict of interest statement

The authors declare that the research was conducted in the absence of any commercial or financial relationships that could be construed as a potential conflict of interest.
